# Hypersensitivity pneumonitis onset and severity is regulated by CD103 dendritic cell expression

**DOI:** 10.1371/journal.pone.0179678

**Published:** 2017-06-19

**Authors:** Emilie Bernatchez, Anick Langlois, Julyanne Brassard, Nicolas Flamand, David Marsolais, Marie-Renée Blanchet

**Affiliations:** Institut Universitaire de Cardiologie et de Pneumologie de Québec, Université Laval, Quebec, Quebec, Canada; Seoul National University College of Pharmacy, REPUBLIC OF KOREA

## Abstract

**Background:**

Pulmonary dendritic cells drive lung responses to foreign antigens, including *Saccharopolyspora rectivirgula*, a causative agent of hypersensitivity pneumonitis. While the airway inflammatory mechanisms involved in hypersensitivity pneumonitis are well described, the mechanisms leading to the break in homeostasis and hypersensitivity pneumonitis onset are not well-described, and could involve CD103^+^ dendritic cells, which are found at baseline and during inflammatory responses in the lung. However, recent demonstration of the ability of CD103^+^ dendritic cells to induce inflammatory responses starkly contrasts with their classically described role as regulatory cells. These discrepancies may be attributable to the lack of current information on the importance of CD103 expression and modulation on these cells during inflammatory episodes.

**Methods:**

To verify the importance of CD103 expression in the regulation of hypersensitivity pneumonitis, wild-type and *Cd103*^*-/-*^ mice were exposed intranasally to *S*. *rectivirgula* and airway inflammation was quantified. Surface expression of CD103 in response to *S*. *rectivirgula* exposure was studied and cell transfers were used to determine the relative importance of CD103 expression on dendritic cells and T cells in regulating the inflammation in hypersensitivity pneumonitis.

**Results:**

*Cd103*^*-/-*^ mice developed an exacerbated inflammatory response as early as 18h following *S*. *rectivirgula* exposure. CD103 expression on dendritic cells was downregulated quickly following *S*. *rectivirgula* exposure, and cell transfers demonstrated that CD103 expression on dendritic cells specifically (and not T cells) regulates the onset and severity of this response.

**Conclusion:**

All in all, we demonstrate that CD103 expression by dendritic cells, but not T cells, is crucial for homeostasis maintenance and the regulation of the T_H_17 airway inflammatory response in hypersensitivity pneumonitis.

## Background

Hypersensitivity pneumonitis (HP) is caused by various antigens, often found in the workplace or home environments [1, 2]. One common form of HP is farmer’s lung disease, caused by exposure to aerosolized *Saccharopolyspora rectivirgula* (SR) antigen, a bacteria found in moldy hay [3]. Recently, high concentrations of SR were also detected in composting plants bioaerosols [4] and associated with the development of HP in workers [5, 6]. This pathology is studied using a mouse model of exposure to an SR antigen preparation, and is defined as a highly polarized T_H_17 inflammatory response [7], where DCs play a major role in disease development and T cells (mainly T_H_17 CD4^+^) are the hallmark of chronic disease severity [7–15]. In addition to T cells, neutrophils are also involved in HP pathogenesis, and are present following acute exposure [16]. While the steps leading to the chronicity of airway inflammation in HP are well described, the mechanisms leading to the break in homeostasis and disease onset in response to HP-inducing antigens are still unknown, impacting our ability to predict sensitization to these antigens and protect workers from developing disease. Additionally, the intrinsic regulatory mechanisms of the inflammatory response to these antigens remain elusive.

The alpha-E integrin CD103-expressing dendritic cells (DCs) and T cells are potential players in regulating the airway inflammatory response in HP. Indeed, CD4/CD103^+^ Tregs possess a stronger suppressive function compared to CD103^-^ counterparts [17] and many studies described a regulatory role for CD103^+^ DCs in a variety of inflammatory contexts in the lung and gut mucosa [18–22]. However, the role of CD103^+^ DCs remains highly controversial, as recent reports alternatively describe this population as either promoting or inhibiting T_H_ responses. Indeed, demonstrations that CD103^+^ DCs induce an exacerbated secretion of pro-inflammatory cytokines by T_H_17 CD4^+^ T cells were recently published [23, 24] while others demonstrated that lung CD103^+^ rapidly produce IL-2 which downregulates IL-17 production by T cells [21]. Furthermore, evidence suggest this specific DC subset primes the T_H_2 response [23–25], while other studies rather propose they fail to sensitize mice to T_H_2 allergens [26], and induce polarization of naïve T cells into Tregs [20]. Therefore, the role of CD103^+^ cells in the development of airway inflammation remains largely unclear.

This may be in part explained by the lack of studies on the specific role of CD103 expression by DC and T cell subsets in airway hypersensitivity disease, which remains unknown. We recently reported that ubiquitous CD103 expression is important in the resolution of airway inflammation in a T_H_2-driven mouse model of asthma [18]; however, whether it is CD103-expressing DCs or T cells that regulates the inflammatory response and whether CD103 can be modulated on DCs and T cells to regulate airway inflammation is currently unknown.

To elucidate this, we used a mouse model of hypersensitivity pneumonitis (HP) caused by exposure to an aerosolized antigenic preparation of *Saccharopolyspora rectivirgula* (SR). Combining the use of *Cd103*^*-/-*^ mice and adoptive DC/T cell transfers [27], we demonstrate that CD103 expression on DCs is reduced at the onset of antigen exposure and that CD103 expression on DCs specifically is important for the regulation of the response onset and to control the magnitude of the inflammatory response to SR. Our study sheds a light on a new mechanism of homeostasis breakdown in airway inflammatory disease and on the crucial role for CD103 expression by DCs in regulating lung inflammatory responses.

## Methods

### Animals

*Cd103*^*-/-*^ (B6.129S2(C)-*Itgae*^*tm1Cmp*^/J), wild type littermates (WT), and *Rag1*^*-/-*^ mice were obtained from Jackson Laboratories and kept in a pathogen-free animal unit (CRIUCPQ; Laval University, Québec, Qc, Canada) for the duration of the experiments.

### Ethics statement

Experiments were approved by local ethics committees and followed Canadian Animal Care guidelines for the use of experimental mice. The study was approved by the Laval University Committee for Animal Care (protocols #2013–124 and #2013–011). Mice were euthanized by an overdose of ketamine/xylazine according to the committee guidelines on rodent euthanasia. No animals died due to the experimental procedures and animals received care to reduce any symptoms of illness or distress when appropriate according to the procedures defined by the Laval University Committee for Animal Care. The condition of the animals was monitored daily.

### Induction of HP and assessment of airway inflammation

**Fig 1:**
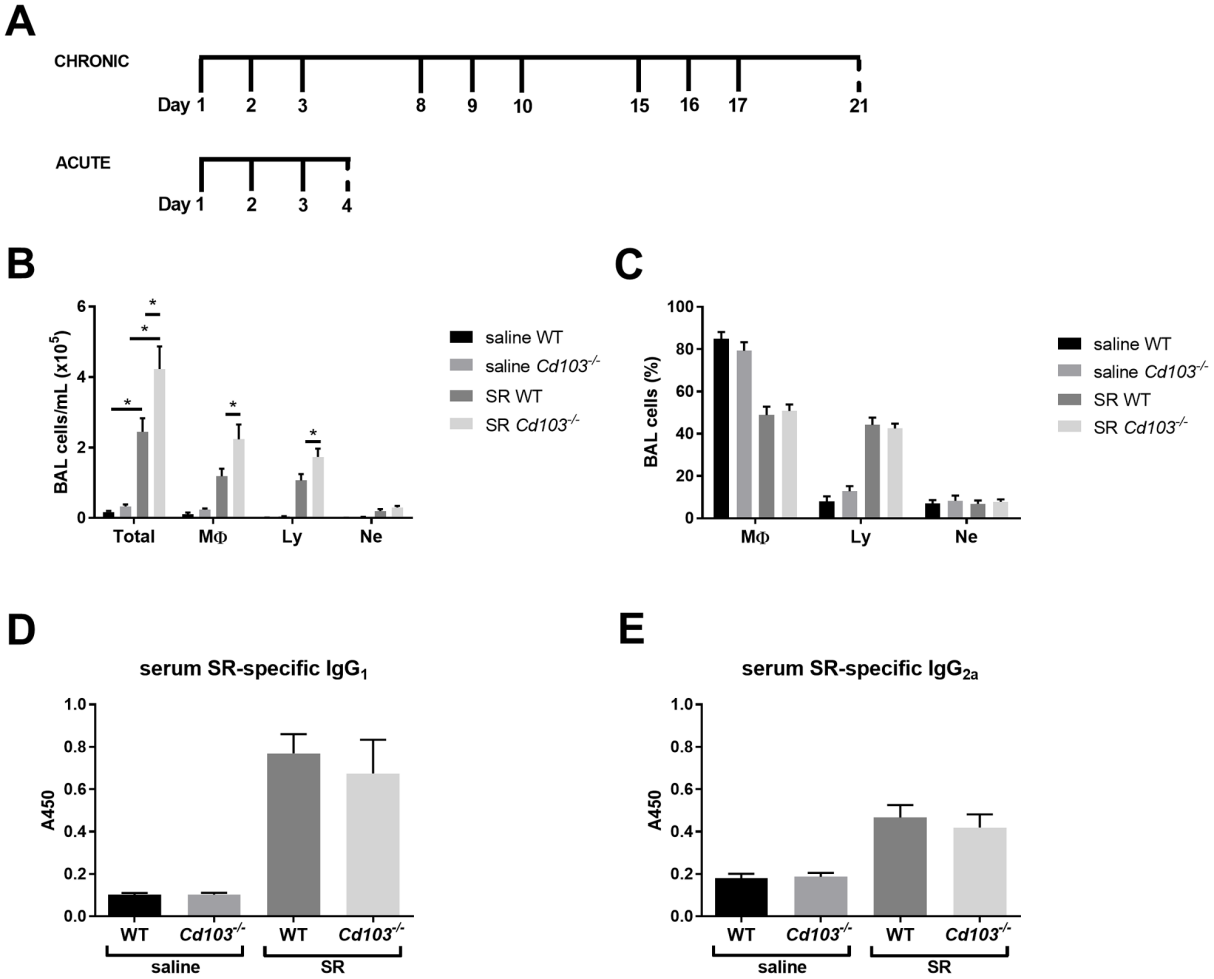
Characterization of the airway inflammatory response in the chronic model of HP: BAL and IgGs. **A)** Timeline of the chronic and acute models of exposure to SR. Full line represents days of intranasal instillation of either saline or SR while dashed line represents day of euthanasia. B-E): WT and *Cd103*^*-/-*^ mice were exposed to SR for 3 weeks and the BAL content and serum immunoglobulins were measured. **B)** BAL total and differential cell numbers (cells/mL) in saline and SR-exposed mice. **C)** BAL differential cell % (MΦ: macrophages, Ly: lymphocytes, Ne: neutrophils) in saline and SR-exposed mice. SR-specific serum levels of **D)** IgG_1_ and **E)** IgG_2a_. For B and C, results were pooled between two experiments, and are representative of over 5 separate experiments; n = 8–12 mice/group. For D and E, results are representative of 2 different experiments; n = 4–6 mice/group. * = p < 0.05.

*Saccharopolyspora rectivirgula* (SR; ATCC 15347) was grown and SR extract was prepared as previously described [28]. The timeline used for the acute and chronic models are presented in Fig 1A. For the chronic model, mice received intranasal instillations of 25μg SR antigen on three consecutive days/week for three weeks. Mice were euthanized 4 days after the last antigen exposure. For the acute model, mice were exposed to 25 ug SR antigen for three consecutive days and euthanized 24h after the last SR exposure. Upon euthanasia, the trachea was cannulated with an 18G catheter, and a broncho-alveolar lavage (BAL) was performed via three separate injections/aspirations of 1mL of saline. Total BAL cells were counted and differential counts obtained using Giemsa stain (HemaStain Set, Fisher Scientific, Kalamazoo, MI). One lung lobe was fixed in 10% formalin for histology studies and hematoxylin/eosin-stained slides were blindly evaluated. A score of 0–5 was attributed for perivascular, peribronchial and parenchymal inflammation, for a total of 15 (0 = no sign of disease, 5 = maximum pathology).

### Detection of antigen-specific IgG_1_ and IgG_2a_

Upon euthanasia, blood was harvested through a cardiac puncture and serum was collected. Briefly, ELISA plates were coated with 50ug/mL of SR antigen, blocked with PBS FBS 10% and incubated with serum dilutions. Samples were then incubated with either, IgG_1_-HRP or IgG_2a_-HRP, followed by revelation with BD OptEIA TMB (BD Biosciences San Diego, CA, USA). The reaction was stopped with 1N HCl.

### Isolation of lung leukocytes

Lung leukocytes were obtained by digestion of lung tissue with Collagenase IV (Sigma, Oakville, ON, Canada) for 0.5hr at 37°C. Digested tissue was pressed through a 70μm cell strainer and leukocytes were enriched using a 30% Percoll gradient (GE Healthcare, Uppsala, Sweden). Lymph nodes were pressed through a 70μm cell strainer. Red blood cells were lysed using ammonium chloride.

### T cell cytokine production assays

CD4^+^ T cell cytokine production was induced by stimulating 1x10^6^ lung-isolated leukocytes with PMA (50ng/mL), ionomycin (750 ng/mL) and brefeldin A (3ug/mL) in RPMI 1640 supplemented with 10% FBS and 1% antibiotic/antimycotic in a 96-well plate for 4 hours. Intracellular cytokine production was analyzed by flow cytometry as described below.

### Flow cytometry analysis of isolated leukocytes

Cells were suspended in PBS supplemented with 10% FBS and 0.02% sodium azide for cell surface staining. Cells were stained with: anti-CD103, anti-CD11c, anti-CD86 (BD Biosciences, Pharmingen, San Diego, CA, USA), anti-CD90.2, anti-CD4, anti-CD8α, anti-MHC-II, anti-XCR1 (BioLegend, San Diego, CA, USA), anti-NK1.1, anti-CD19 (Ablab, Vancouver, B.C., Canada), anti-CD11b (eBioscience, San Diego, CA, USA). For intracellular staining, cells were fixed and permeabilized using the Intracellular Fixation & Permeabilization Buffer Set (eBioscience). Antibodies used were: anti-IL-13 (eBioscience), anti-IFNg, and anti-IL-17A (BioLegend).

### DC in vivo expansion and in vitro stimulation

For *in vivo* DC expansion and isolation, WT mice were subcutaneously injected in the lower back with 5x10^5^ Flt3 ligand-producing B16 melanoma cells, previously grown in DMEM media supplemented with 10% FBS and 1% antibiotic/antimycotic. When the tumor reached 1cm diameter, mice were euthanized and the lung and spleen collected. Total lung leukocytes were harvested as described above. Total spleen leukocytes were harvested by pressing through a 70μm strainer followed by a red blood cells lysis with ammonium chloride. DCs were purified by negative selection using the EasySep Mouse pan-DC Enrichment Kit (StemCell Technologies, Vancouver, BC, Canada). 3x10^5^ isolated DCs/ml were stimulated with SR (final concentration of 0, 1 or 5μg/mL) in a DC-specific complete media (RPMI 1640, 10% FBS, 10% GM-CSF (UBC Ablab, Vancouver, BC, Canada), 1% antibiotic/antimycotic, 1% sodium pyruvate, and 40 μM β-mercaptoethanol) for 18h. Following SR stimulation, cells were counted using trypan blue.

### RNA isolation and quantitative PCR

RNA samples were prepared using EZ-10 DNAaway RNA Mini-Preps kit (BioBasic Canana INC., Markham, CA) and cDNA was synthesized using iScript cDNA Synthesis Kit (Bio-rad, Mississauga, CA). Real-time PCR was performed using SsoAdvenced Universa SYBR Green Supermix (Bio-rad) and a rotor gene 6000 (QIAGEN, Valencia, Calif). Primers for quantitative PCR: *Itgae* (CD103): 5’-AGGTCATAGATACGGTCAGGT-3’ (fwd) and 5’-GGTTAGATTTCAATGGCGATGG-3’ (rev). Values were normalized to the expression of Rplp0 and GNB for each sample. Mean relative gene expression was determined and the differences were calculated using the 2^-ΔC(t)^ method.

### DC and T cell transfers

For DC transfers, 1x10^6^ DCs (isolated as described above) were injected intravenously into *Cd103*^*-/-*^ or WT mice in a criss-cross manner 24 hours before the first exposure to SR [29].

For T cell transfers, lymph nodes were collected from naïve WT or *Cd103*^*-/-*^ mice and leukocytes were harvested as described above. CD4^+^ cells were purified using EasySep Mouse CD4^+^ Enrichment Kit (StemCell Technologies). 0.5x10^6^ isolated CD4^+^ T cells were injected intravenously into *Rag1*^*-/-*^ mice 1 month before the first exposure to SR as described [30].

### Statistics

Data are presented as mean ± SEM. Data was tested for normality and homogeneity of variance. Statistical analysis for multiple comparisons was performed using an ANOVA table followed by Tukey’s multiple comparison test. Non-multiple comparisons were analyzed using unpaired T-tests. Statistical significance was determined at p < 0.05.

## Results

### Lack of CD103 expression exacerbates the inflammatory response in HP

**Fig 2:**
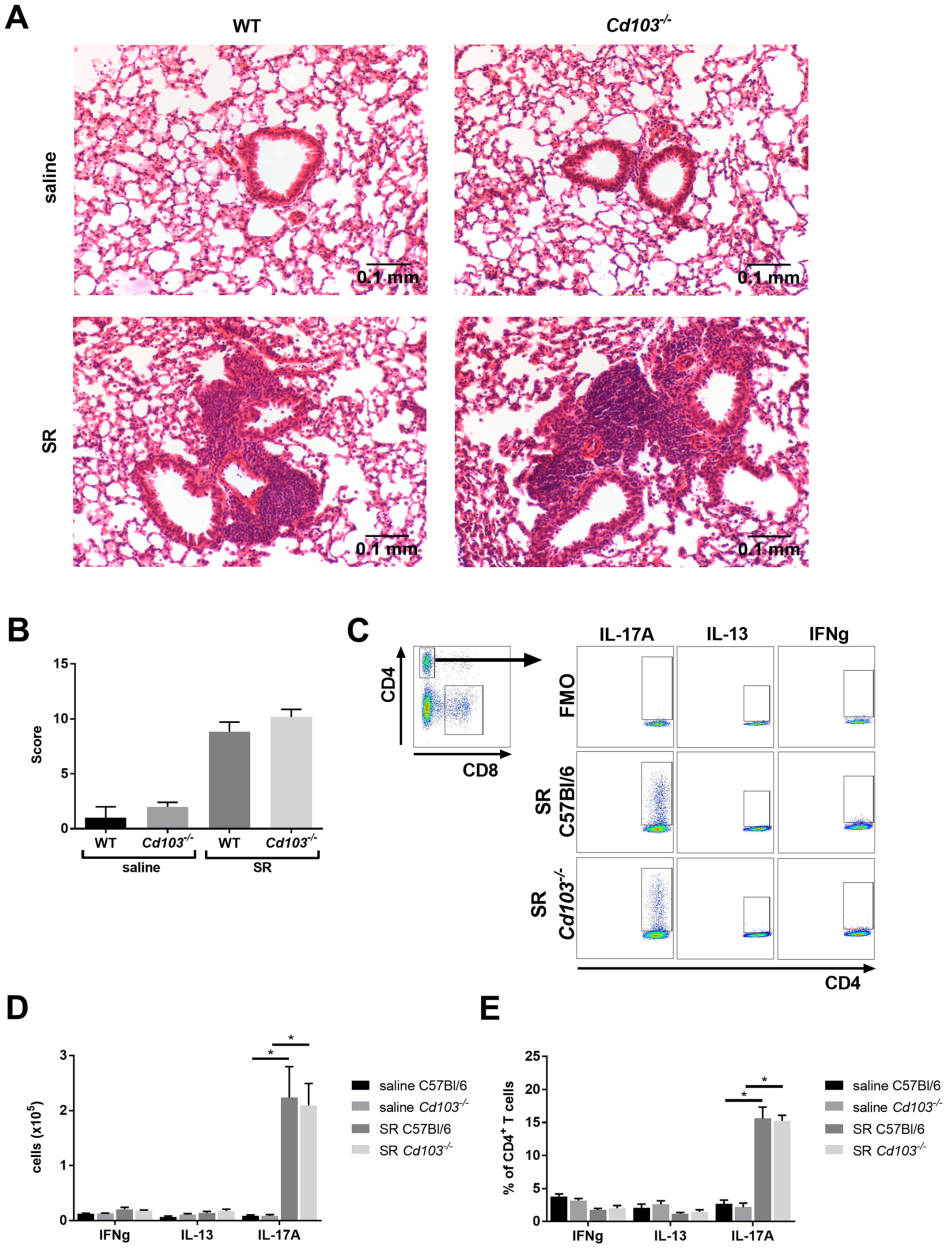
Characterization of the airway inflammatory response in the chronic model of HP: Histology and cytokine. WT and *Cd103*^*-/-*^ mice were exposed to SR for 3 weeks and a section of the left lobe was used for histology while other lobes were used for analysis of cytokine production. **A)** Lung sections of WT and *Cd103*^*-/-*^ mice exposed to saline or SR. **B)** Histological score was obtained and compared between WT and *Cd103*^*-/-*^ mice. **C)** Flow cytometry gating strategy for the polarity of the effector lung response after *ex vivo* stimulation of lung leukocytes isolated from SR-exposed mice. CD4^+^ T cells were gated from total lung cells and cytokine-positive cells were analyzed using Fluorescence Minus One (FMO) controls. **D)** Number of cells and **E)** % of IFNg^+^, IL-13^+^ and IL-17A^+^CD4^+^ T cells in the lung of saline and SR-exposed mice. Results representative of 2 different experiments; n = 4–6 mice/group. * = p < 0.05.

The regulatory mechanisms of T_H_17 airway hypersensitivity inflammatory responses, such as in HP, are not well understood. Although it is suggested that CD103^+^ DCs control the intensity of this adaptive immune response by limiting the expansion of T_H_17 CD4^+^ T cells [21], other reports suggest that these cells prime this specific T cell response [23, 24]. Additionally, the role of CD103 expression on this DC subset remains unknown. Therefore, we first set out to verify whether the absence of CD103 expression influences the lung inflammatory response in chronic HP. As noted by the increased number of total inflammatory broncho-alveolar lavage (BAL) cells, lymphocytes and alveolar macrophages, *Cd103*^*-/-*^ mice developed an exacerbated total inflammatory response to SR antigen compared to wild-type (WT) controls (Fig 1B). Interestingly, neither the % of BAL cell subsets nor the IgG1/IgG2a production differed between SR antigen-exposed *Cd103*^*-/-*^ and WT mice (Fig 1C–1E). When looking at lung histology, no difference was observed in the perivascular, peribronchial and parenchymal inflammation between both strains following SR exposure (Fig 2A and 2B), suggesting that in our model, only the severity of the airway inflammation / alveolitis is regulated by CD103 expression, which is the major hallmark of this specific pathology.

We then set out to verify whether CD103 expression regulates T cell polarity, which could partially explain the exacerbated HP response in *Cd103*^*-/-*^ mice. To evaluate this, CD4^+^ T cell polarity was measured via IFN-g (T_H_1 response), IL-13 (T_H_2 response) and IL-17A (T_H_17 response) intracellular cytokine production. As previously described, the T cell response following SR administration was largely T_H_17-driven, as seen by the high increase of IL-17A^+^ CD4^+^ T cells in WT mice but not of IFN-g^+^ CD4^+^ and IL-13^+^ CD4^+^ T cells (Fig 2D and 2E). Interestingly, no difference was observed in the CD4 polarity between WT and *Cd103*^*-/-*^ mice (Fig 2D and 2E). These results indicate that in our model, CD103 expression does not affect the type and polarity of the inflammatory response, and suggest a role for CD103 in regulating other mechanisms of HP pathophysiology and disease severity.

### CD103 expression regulates the early onset of HP

**Fig 3:**
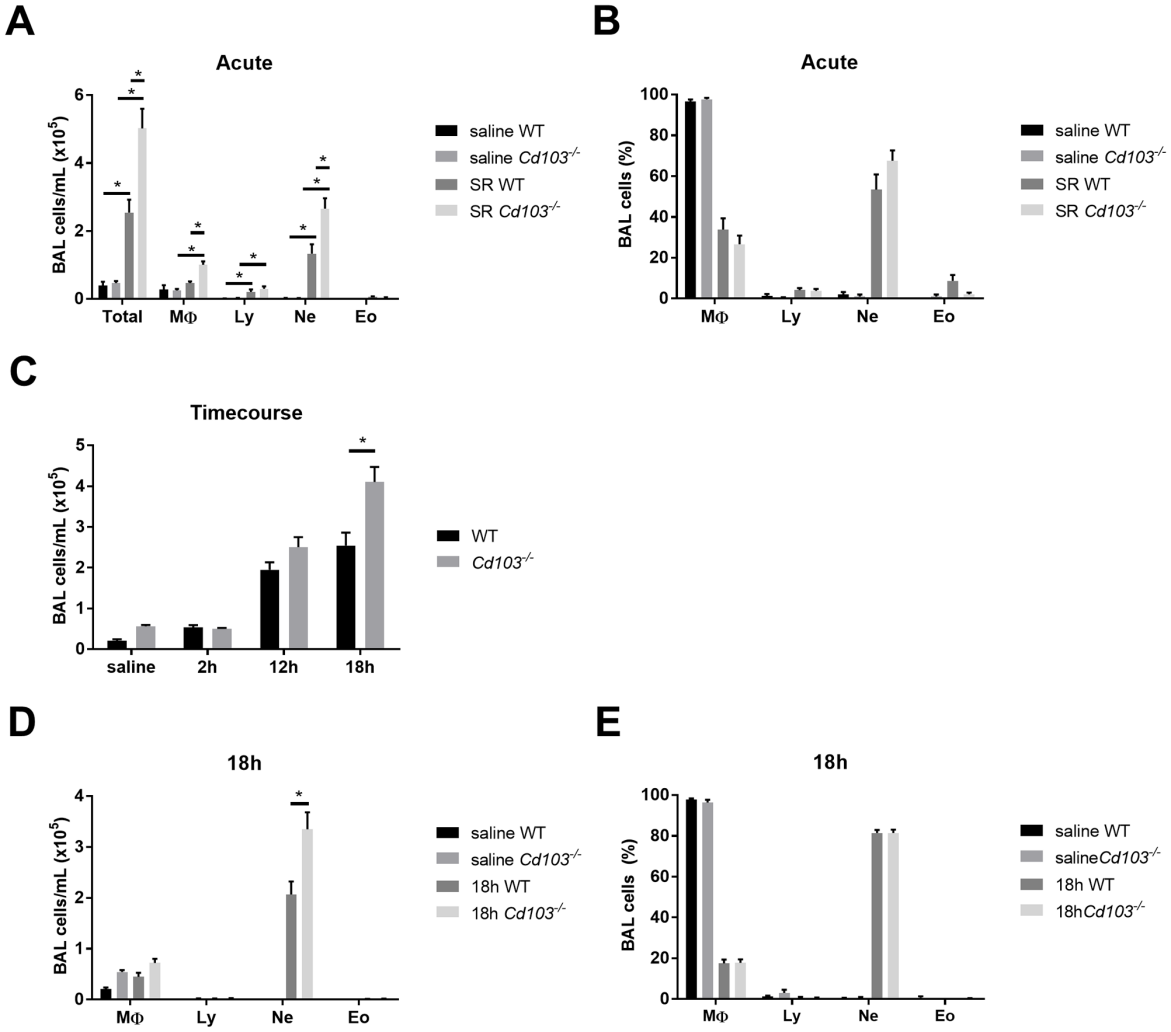
Airway inflammatory response in WT and *Cd103*^*-/-*^ mice in the acute model of HP. WT and *Cd103*^*-/-*^ mice were exposed to SR for 3 days and the BAL content was evaluated: **A)** BAL total and differential cell numbers (cells/mL) in saline and SR-exposed mice; **B)** BAL differential cell % (MΦ: macrophages, Ly: lymphocytes, Ne: neutrophils, Eo: eosinophils) in saline and SR-exposed mice. C-E): WT and *Cd103*^*-/-*^ mice were exposed to SR and the BAL content was evaluated 2h, 12h and 18h after the exposure: **C)** BAL total numbers (cells/mL) in saline and SR-exposed mice; **D)** BAL differential numbers (cells/mL) and **E)** % 18h after exposure to saline or SR. For A and B, results from 2 experiments were pooled; n = 6–12 mice/group. For C and D, results are representative of 2 different experiments; n = 3–4 mice/group. * = p < 0.05.

Results in the chronic model confirm that CD103 expression is important for the regulation of the airway inflammatory response, as we have previously observed in asthma [18]. However, we observed that CD103 expression does not impact T cell polarization (Fig 2D and 2E); therefore, CD103 expression likely regulates other mechanisms of disease pathophysiology. As Zelante *et al*. suggested that rapid production of IL-2 by CD103^+^ DCs is important for the control of the inflammatory response [21], we investigated the role for CD103 expression in the initiation of the inflammatory response in HP. Following an acute (3 days) exposure to SR, *Cd103*^*-/-*^ mice developed an increased inflammatory response compared to WT mice, characterized by increased neutrophil infiltration into the airways (Fig 3A). Moreover, again, the type of inflammatory response was not affected in the absence of CD103 expression, with neutrophils proportions similar between both strains (Fig 3B). This indicates that CD103 plays a role in controlling the intensity of the early immune response to SR, rather than dictating the response polarity. To further discern the role of CD103 in the early response to SR, WT and *Cd103*^*-/-*^ mice were euthanized at 2h, 12h, or 18h after a single exposure to SR. Interestingly, the exacerbated inflammatory response in *Cd103*^*-/-*^ mice was observed as early as 18 hours after the first exposure to SR, and was characterized by increased neutrophils (Fig 3C–3E), suggesting an important role for CD103 expression in the control of the early onset of the inflammatory response.

### CD103 expression on DCs is downregulated at the onset of the response to SR

**Fig 4:**
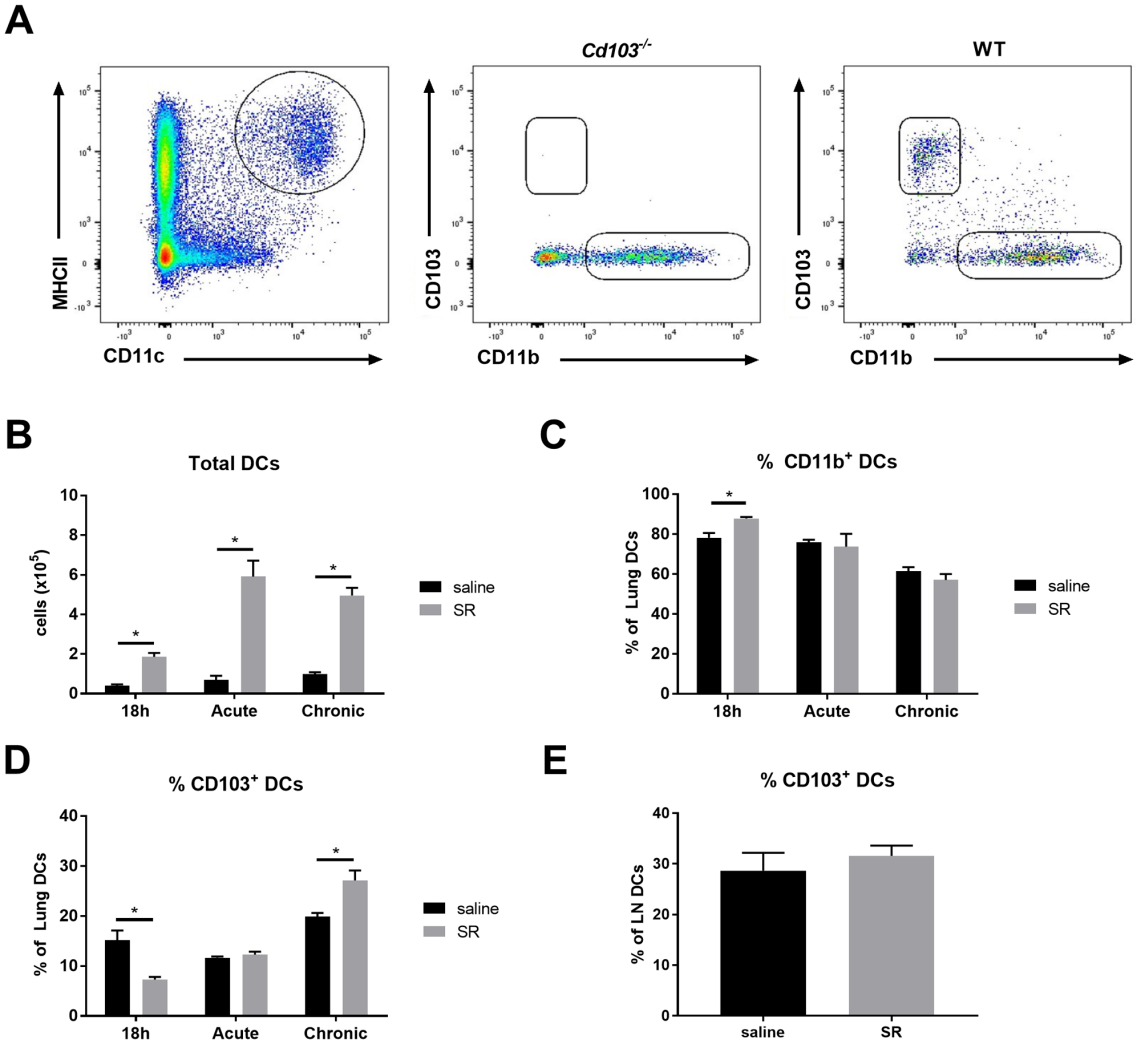
Characterization of lung DCs populations in response to SR. DCs were characterized 18h following SR exposure, in the acute and in the chronic model. **A)** Flow cytometry gating strategy of DCs populations obtained after lung harvest. DCs were identified as autofluorescence^-^ / NK1.1^-^, CD90.2^-^, CD19^-^ / CD11c^+^/ MHC-II^hi^ cells. CD103 expression on DCs was verified in WT samples using *Cd103*^*-/-*^ mice as control for CD103 staining. **B)** Total lung DCs. % of **C)** CD11b^+^ and **D)** CD103^+^ lung DCs. **E)** % of CD103^+^ mediastinal LN DCs. Results are representative of 2 separate experiments; n = 4–6 mice/group. * = p < 0.05.

To further discern how CD103 expression plays a role in the regulation of the inflammatory response, we characterized DC populations in the early onset, acute and chronic exposure to SR. We observed that total DCs are increased as early as 18h after the first SR exposure and through acute and chronic responses (Fig 4B). However, when looking at the % of cells, we observed a slight increase in the % of CD11b^+^ DCs (generally characterized as pro-inflammatory) 18h following SR exposure (Fig 4C). In opposition to CD11b^+^ DCs, the % of CD103^+^ DCs is significantly decreased in WT mice at this time point (Fig 4D). To verify whether this could be explained by increased CD103^+^ DCs migration to the lymph nodes (LN), the % of CD103^+^ DCs in the mediastinal LN was analyzed; no increase in this cell population was observed following SR exposure (Fig 4E).

**Fig 5:**
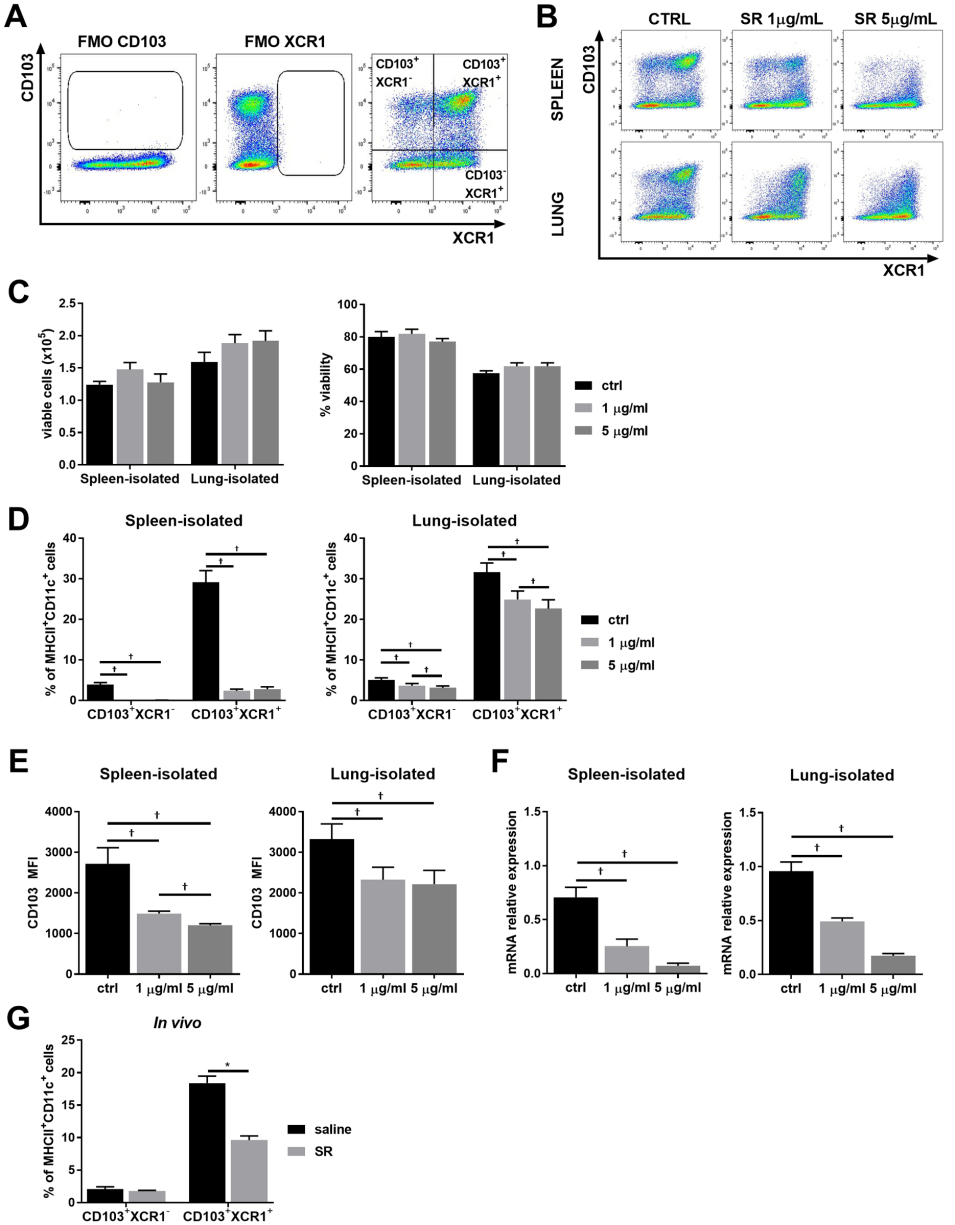
Modulation of CD103 expression on DCs in response to SR *in vitro*. DCs were isolated from lung and spleen of WT mice and stimulated with 0μg/mL (ctrl), 1μg/mL or 5μg/mL of SR extract for 18h. DCs were identified as autofluorescence^-^/ CD11c^+^/ MHC-II^hi^ cells and CD103 and XCR1 expression was analyzed as shown in **A)**. **B)** Flow cytometry examples of CD103 and XCR1 expression on SR-stimulated spleen- and lung-isolated DCs. **C)** Viability, measured with trypan blue, of spleen- and lung-isolated DCs after stimulation with SR. **D)** % of spleen- and lung-isolated DCs expressing CD103 (CD103^+^XCR1^-^) or XCR1 and CD103 (CD103^+^XCR1^+^). **E)** Mean Fluorescence Intensity (MFI) of CD103 from spleen- and lung-isolated CD103^+^XCR1^+^ DCs. **F)** Transcript level of CD103 relative to Rplp0 and GNB in spleen- and lung-isolated DCs. **G)** *In vivo* CD103^+^ or CD103^+^XCR1^+^ on lung CD11c^+^/ MHC-II^hi^ DCs (as analyzed in Fig 4A) 18h after SR exposure. Results are representative of 2 separate experiments; n = 5–6 mice/group. * = p < 0.05. † = p < 0.05 with multi-comparison test.

Alternatively, the reduced % of CD103^+^ DCs could be explained by a direct impact of SR exposure on CD103 expression by DC populations. We thus verified *in vitro* the changes in DC molecule expression pattern in response to SR antigen. To answer this, spleen and lung DCs were isolated from mice injected with Flt3-ligand producing melanoma (to expand the DC subsets in these organs) as previously described [31]. Cells were stimulated *ex-vivo* with SR antigen, and, as XCR1 and CD103 are co-expressed markers on DCs [32, 33], the % of CD103^+^XCR1^-^ and CD103^+^XCR1^+^ DCs as well as CD103 mean fluorescence unit (MFI; indicative of total surface protein expression) were evaluated. The # and viability of spleen- and lung-isolated cells were similar between control (ctrl) and stimulated cells (Fig 5C). Data shows that the % of CD103^+^XCR1^-^ and CD103^+^XCR1^+^ cells (Fig 5D), total CD103 surface expression (Fig 5E), as well as CD103 mRNA (Fig 5F) was significantly reduced in spleen- and lung-isolated cell cultures following SR stimulation. This was also observed *in vivo*, where most cells are CD103^+^XCR1^+^ and this population is significantly decreased 18h following SR exposure (Fig 5G). These important results demonstrate that the decrease in % of CD103^+^ DCs observed 18h after SR exposure could be due to a direct downregulating effect of SR on CD103 DC expression. Modulation of CD103 expression directly on DCs could therefore be an important mechanism by which SR breaks lung homeostasis and onsets the airway hypersensitivity response in HP.

### Modulation of CD103 expression on T cells and CD103 in Tregs population maintenance

**Fig 6:**
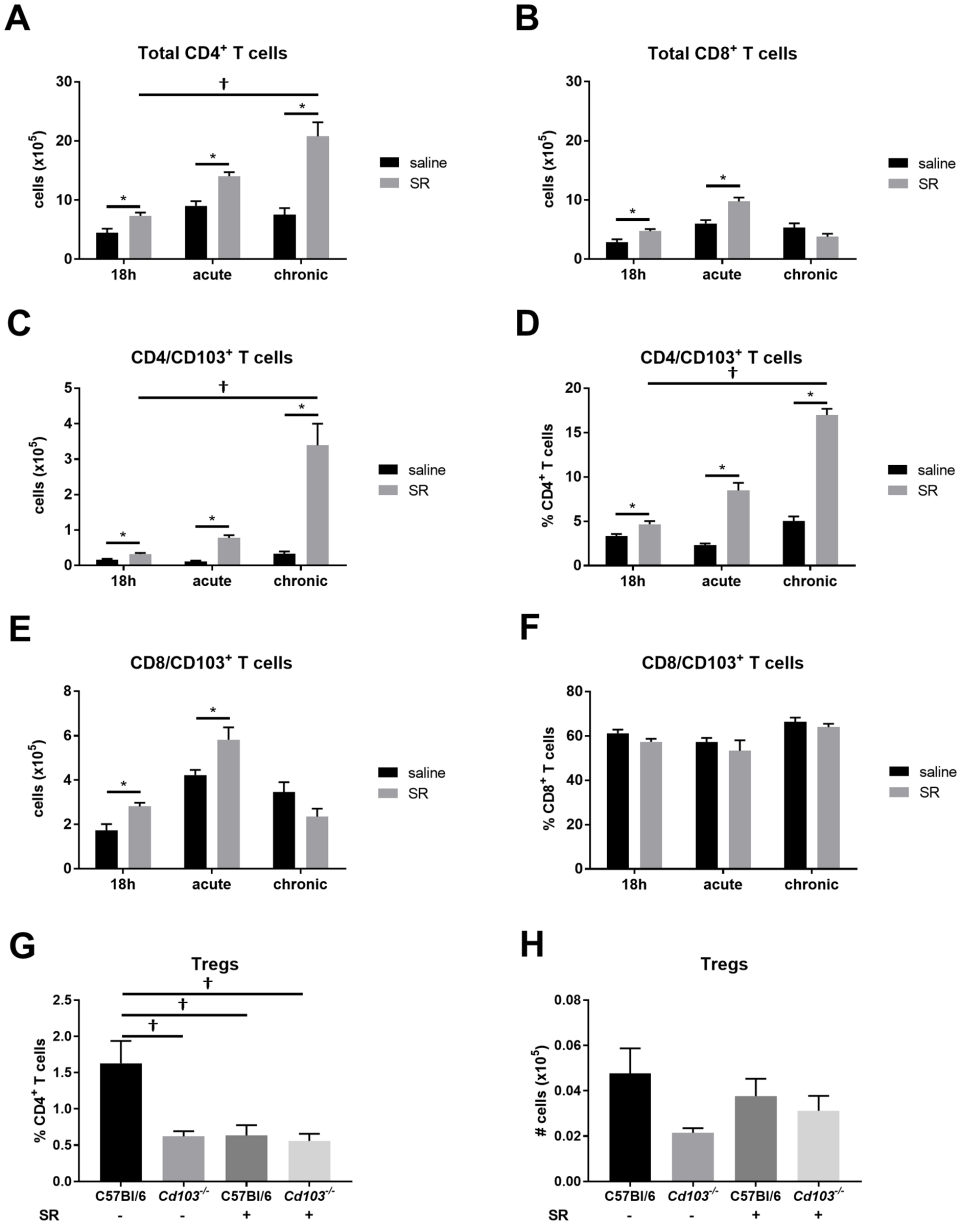
Characterization of T cells populations in response to SR. T cells were characterized after 18h, in the acute and in the chronic model. They were identified as CD4^+^ or CD8^+^, then CD103 expression on T cell subsets was verified in WT samples using *Cd103*^*-/-*^ mice as control for CD103 staining. Number of total lung **A)** CD4^+^ T cells and **B)** CD8^+^ T cells. **C)** Number and **D)** % of CD4/CD103^+^ T cells. **E)** Number and **F)** % of CD8/CD103^+^ T cells. For Tregs analysis CD25^+^Foxp3^+^ on lung CD4^+^ T cells was verified using FMO controls. **G)** % and **H)** number of Tregs. Results were obtained from 2 pooled experiments; n = 6–12 mice/group. * = p < 0.05 for unpaired t-test. † = p < 0.05 with multi-comparison test.

To assess the importance of CD103 expression on T cells, we first characterized CD103^+^ T cell populations following SR administration. In our hands, the general T cell response to SR was characterized by a large increase in total CD4^+^ T cells (vs CD8^+^ T cells) (Fig 6A and 6B). In addition, CD4^+^/CD103^+^ T cells number and % are increased in response to SR, with the most drastic increase observed in the chronic response (Fig 6C and 6D). In comparison, the increase in the CD8^+^/CD103^+^ T cell population is observed earlier in the time course (up to the acute response) and returns to basal level in the chronic response (Fig 6E and 6F). This important increase of CD4^+^ and of CD4^+^/CD103^+^ T cells in the lung of chronically SR-exposed mice suggest a role for this population in the ongoing T_H_17 inflammatory response, whilst the CD8^+^/CD103^+^ population could play an earlier role in the response to SR.

Additionally, since CD103^+^ DCs play a role in Tregs induction [19, 20, 22], we verified whether the exacerbated observed lymphocytosis in the absence of CD103 expression was caused by alteration of Tregs in *Cd103*^*-/-*^ mice. To do this, we looked at CD4/CD25^+^Foxp3^+^ T cells in the lung of WT and *Cd103*^*-/-*^ mice. We observed decreased Tregs % in the lung of saline-exposed *Cd103*^*-/-*^ mice compared to WT (Fig 6G). Following SR exposure, the % of Tregs in the lung is reduced in WT mice compared to saline (Fig 6G), likely due to the increased CD4 T cell response and severe lymphocytosis described above (Fig 6G). The % of Tregs in *Cd103*^*-/-*^ mice is not different compared to their controls (Fig 6G). Moreover, in our hands, no significant changes in the number of Tregs were observed between strains, or following antigen exposure (Fig 6H).

### CD103 expression on DCs specifically regulates the early onset and ongoing inflammatory response

**Fig 7:**
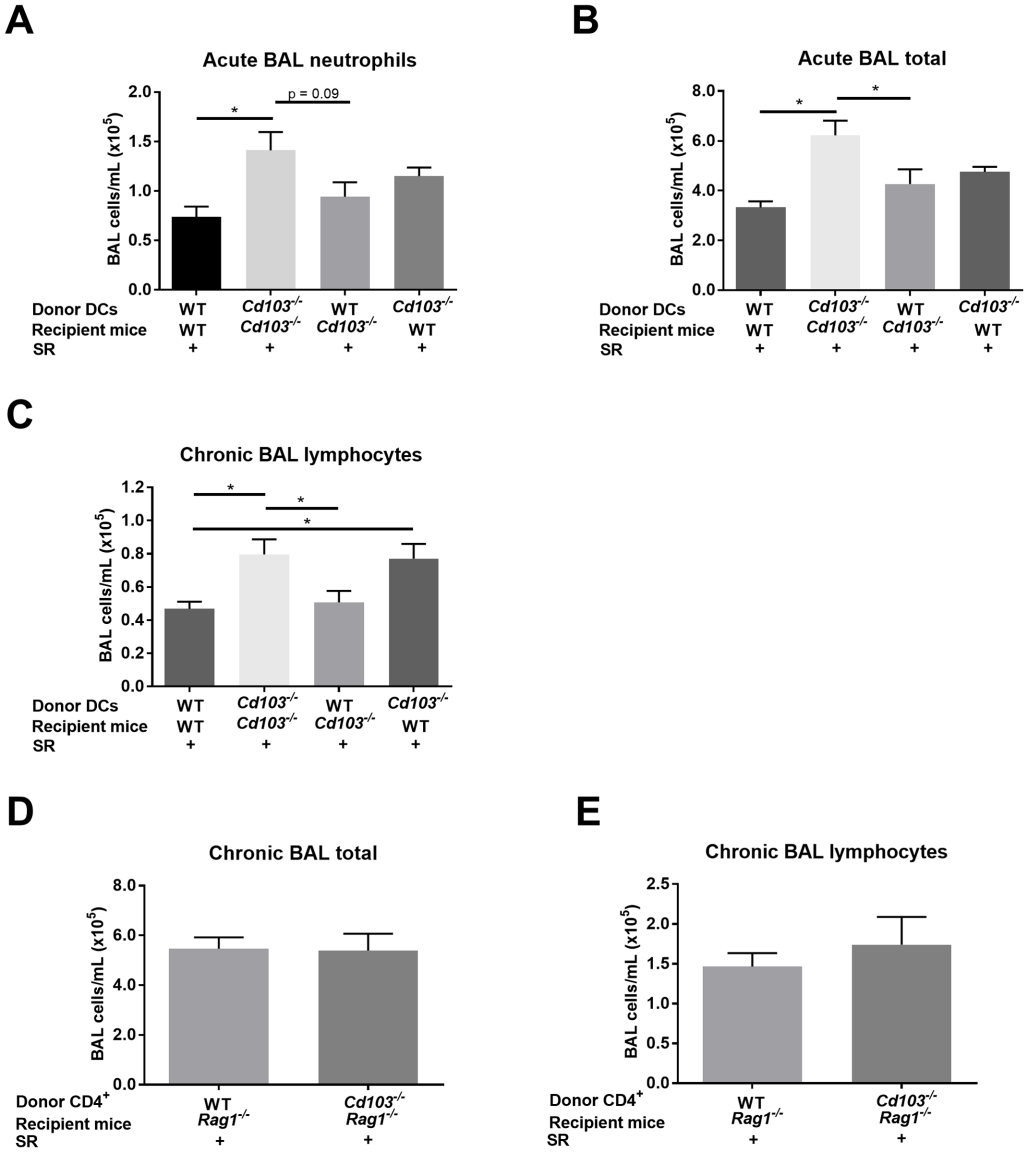
Transfers of DCs and CD4^+^ T cells. WT and *Cd103*^*-/-*^ DCs were injected in a criss-cross manner in WT and *Cd103*^*-/-*^ mice. 24h after injection, recipient mice were submitted to either the acute or chronic model. **A)** Total BAL neutrophils (cells/mL) and **B)** total BAL cells (cells/mL) of SR-exposed mice in the acute model. **C)** Total BAL lymphocytes (cells/mL) of SR-exposed mice in the chronic model. WT or *Cd103*^*-/-*^ CD4^+^ T cells were injected in *Rag*^*-/-*^ mice. 1 month after the transfer, recipient mice were submitted to the chronic model. **D)** Total BAL cells (cells/mL) and **E)** total BAL lymphocytes (cells/mL) of saline and SR-exposed mice. Results were pooled from two experiments; n = 6–10 mice/group. * = p < 0.05 with multi-comparison test.

Our results show that lack of CD103 expression affects the inflammatory response at the onset of antigen exposure. Additionally, Zelante *et al*. show that rapid secretion of IL-2 by CD103^+^ DCs is important in controlling the severity of the lung inflammatory response [21]. However, whether CD103 expression by DCs specifically at the onset of antigen exposure is necessary to control the ongoing inflammatory response remains unknown. To assess this, one injection of WT or *Cd103*^*-/-*^ DCs was done in a criss-cross manner (WT into WT, WT into *Cd103*^*-/-*^, and vice-versa) 10h before the first exposure to SR. This technique has been used to study the role of DCs in lung responses [29, 34, 35] and we and others [36] have been able to find transferred DCs in the lung after injection. Following DC injection, the acute and chronic SR models were developed in DC-transferred mice and the severity of the inflammatory response was evaluated. In the acute model, we found that a single administration of WT DCs into *Cd103*^*-/-*^ mice reduces the total inflammatory response as well as neutrophilia back to WT levels (Fig 7A and 7B). To verify whether this effect would stand in time, the same experiment was reproduced in the chronic model. Surprisingly, we found that a single injection of WT DCs into *Cd103*^*-/-*^ mice 10h before the first SR exposure reduced the lymphocytosis (the principal hallmark of chronic HP) back to WT levels (Fig 7C). Of note, the injection of *Cd103*^*-/-*^ DCs into WT mice exacerbated the lymphocytosis observed in the chronic model. These results confirm the crucial role for CD103 expression on DCs in the control of the onset of this T_H_17 inflammatory response, which ultimately regulates the ongoing lymphocytosis.

As the role for CD4^+^/CD103^+^ T cells in the lung is not well understood and as CD4^+^/CD103^+^ T cells are increased in our model, we verified the importance of CD103 expression on CD4^+^ T cells in the ongoing inflammatory response in HP. To do so, CD4^+^ T cells isolated from lymph nodes of either WT of *Cd103*^*-/-*^ mice were injected intravenously in *Rag1*^*-/-*^ mice. Results revealed that CD103 expression on CD4^+^ T cells did not affect the inflammatory response to SR (Fig 7D and 7E); thus, CD103 expression by CD4^+^ T cells is not necessary for control of the ongoing inflammatory response.

## Discussion

CD103 expression on DCs and T cells was studied in many models, but the exact role of CD103 expression in disease (beyond its integrin activity) is not well defined. In the lung, CD103^+^ DCs are alternatively associated with the induction of airway inflammation [23–25, 37], and regulatory mechanisms of airway inflammation [18, 20, 21]. The discrepancies in literature may be attributed to the lack of studies on the importance and timeline of CD103 expression on DCs in hypersensitivity lung responses such as in HP. While the steps leading to the chronicity of the inflammatory response in HP are well-described, the lack of information on the mechanisms regulating the initiation of disease impact our ability to predict sensitivity to HP-inducing antigens in workers, which is a considerable need in the fight against professional hypersensitivity airway disease. Here, we clearly demonstrate that CD103 expression regulates the intensity of the onset inflammatory lung response following the initial exposure to an antigenic preparation of SR. Indeed, lack of CD103 expression leads to an exacerbated inflammatory response as early as 18 hours after antigen exposure that is maintained in time. While the exacerbated early neutrophil response might explain the increased chronic lymphocytosis in *Cd103*^*-/-*^ mice, the exact mechanism by which CD103 expression regulates the severity of neutrophil recruitment at the onset of the airway response to SR remains unknown.

DCs are the first line of defense in the lung. Resident DCs (CD103^+^ and CD11b^+^) migrate to afferent lymph nodes for T cell polarization in response to antigens, while circulating DCs are rapidly recruited to the lung. Interestingly, in our model, the proportion of CD103^+^ DCs is decreased in the lung 18 hours following the first antigen exposure. Studies in the skin, gut and lung demonstrated that CD103^+^ DCs can migrate to the lymph nodes after capturing antigens [38–40]. At first sight, this could explain our observations. However, we did not observe an increase of this population in the mediastinal lymph nodes following SR exposure. Rather, we demonstrate that exposure to SR antigen *in vitro* leads to a reduction of CD103 protein and mRNA expression in DCs. Rapid apoptosis of DCs could also be another factor leading to the decrease in CD103^+^ DCs. However, no decrease of cell number or viability was observed following stimulation, indicating that alterations in cell viability cannot explain this result. Whilst we can not rule out that the decrease in CD103^+^ DCs *in vivo* following SR exposure could be due to a more robust recruitment of CD11b^+^ DCs over CD103^+^ DCs, our results *in vitro* demonstrating a clear reduction of CD103 expression on DCs following SR stimulation explain, at least in part, the observed SR-induced *in vivo* decrease in CD103^+^ DCs. Importantly, loss of CD103 expression following the initial antigen exposure may lead to intrinsic changes in DC function. Whether this would have consequences on the subsequent inflammatory response requires further investigation.

Independently of the dampening effect of SR exposure on CD103 expression on DCs, our results show that the small population of CD103^+^ DCs that remains at baseline 18h after exposure to SR is crucial for regulating the severity of inflammatory response in the lung. This is first supported by the exacerbated initial inflammatory response in the absence of CD103, and confirmed by DC transfer experiments. Indeed, a single administration of WT DCs in *Cd103*^*-/-*^ mice, which provides some CD103 expression by DCs in *Cd103*^*-/-*^ mice at the time of first SR exposure, reverses the exacerbation phenotype observed in these mice up to 3 weeks following exposure. This initial process might be IL-2 dependent, as observed by Zelante, *et al* [21]. This observation and our previous report on the importance of CD103 expression for the initiation of resolution processes suggests a time-dependant importance of CD103 expression in regulating airway inflammation. This could also explain the increase in CD103^+^ DCs in the chronic model, where CD103 expression by DCs could participate in the resolution of airway inflammation in response to SR [18]. Furthermore, the exacerbated phenotype in *Cd103*^*-/-*^ mice could partly be explained by the observed reduced % of Tregs in the lung at basal level, which supports previous reports demonstrating that CD103^+^ DCs favour the differentiation of T naïve cells into Tregs [19, 20, 22]. However, we see no increase in Tregs in WT or *Cd103*^*-/-*^ mice after SR exposure. Other studies have analyzed Tregs in response to SR and found an increase of this cell population in the lung [41, 42], but their model uses higher quantities of SR, which could explain the discrepancies between our studies. Our results therefore suggest that CD103 expression does not impact the Tregs response to SR antigen and that the exacerbation phenotype is not caused by a dysregulation of Tregs in *Cd103*^*-/-*^ mice.

Results from transfer models also pin pointed that CD103 expression by DCs specifically, and not by CD4^+^ T cells, is required for control of the ongoing inflammatory response to SR. Indeed, based on our result in *Rag1*^*-/-*^ mice, CD103 expression specifically on CD4^+^ T cells is not required for the regulation of the inflammatory response to SR. Although the CD4^+^/CD103^+^ T cell population expands greatly in our model, the importance of CD103 expression on CD4^+^ T cells in lung responses remains elusive. However, our results are in agreement with studies implying that CD103 expression only on non-T cells is essential for the regulatory role of CD103^+^ cells in the gut [43].

A natural hypothesis to explain the exacerbation phenotype observed in *Cd103*^*-/-*^ mice is that this strain possesses an intrinsic pro-inflammatory environment that supports more severe inflammatory reactions to antigens. However, we clearly demonstrate that the balance of T_H_17 and T_H_1-producing CD4^+^ T cells does not differ in this model. Moreover, the injection of WT DCs reverses the phenotype in *Cd103*^*-/-*^ mice, indicating that the *Cd103*^*-/-*^ background is likely not the reason for the exacerbation observed in these mice. Another hypothesis could be that *Cd103*^*-/-*^ DCs may possess a different intrinsic inflammatory activity, as it was observed that injection of *Cd103*^*-/-*^ DCs exacerbates the lymphocyte response in WT mice in the chronic model. However, when interpreting results in DCs transfer experiments, it is important to keep in mind that the lung is likely flooded with the injected DCs. Consequently, and rather than suggesting an intrinsic inflammatory defect in *Cd103*^*-/-*^ DCs, our results strongly suggest that the incapacity of DCs to express CD103 at the time of disease induction is a major factor in the inflammation exacerbation in these mice, reinforcing the importance of CD103 expression on DCs specifically in the regulation of T_H_17 responses.

## Conclusions

All in all, our data clearly demonstrate that CD103 expression on DCs, and not CD4^+^ T cells, at the onset of the inflammatory response is crucial in the regulation of the severity of the inflammatory response in HP. This unravels a new and important mechanism for CD103 expression on DCs specifically in the breakdown of homeostasis following exposure to SR antigen in HP. Further studies on whether exposure to other antigens also dampens CD103 expression on DCs and how it affects the initiation of the inflammatory cascade need to be conducted.
